# Longitudinal changes in optic disc cupping from the baseline in chiasmal lesion optic neuropathy and glaucoma

**DOI:** 10.1038/s41598-024-59419-3

**Published:** 2024-04-17

**Authors:** Eun Jung Lee, Jong Chul Han, Mira Kang, Doo-Sik Kong, Sang Duk Hong, Kyung-Ah Park, Changwon Kee

**Affiliations:** 1 Department of Ophthalmology, Samsung Medical Center, Sungkyunkwan University School of Medicine, Seoul, 06351 Korea; 2 Center for Health Promotion, Digital Innovation Center, Samsung Medical Center, Seoul, Korea; 3 Department of Neurosurgery, Samsung Medical Center, Sungkyunkwan University School of Medicine, Seoul, Korea; 4 Department of Otolaryngology, Samsung Medical Center, Sungkyunkwan University School of Medicine, Seoul, Korea; 5 Department of Ophthalmology, Kim’s Eye Hospital, Konyang University College of Medicine, Seoul, Korea

**Keywords:** Diseases, Eye diseases, Optic nerve diseases

## Abstract

We aimed to investigate the changes in cupping in chiasmal lesion optic neuropathy (chON) compared to baseline optic disc and glaucoma. We used a novel study design to enroll patients who had fundus photographs incidentally taken during routine health check-ups prior to the onset of optic neuropathy. In 31 eyes (21 patients) with chON and 33 eyes (30 patients) with glaucoma, we investigated the change in cup-to-disc (C/D) area from the baseline to overt cupping using flicker analysis. Compared to the baseline, 23 eyes (74.2%) had increased cup size and 3 (9.7%) had vascular configuration changes in the chONgroup; in contrast, all glaucoma eyes exhibited changes in cup size and vascular configuration. The increase in C/D area ratio was significantly smaller in chON (0.04 ± 0.04) compared to glaucoma (0.10 ± 0.04, *P* < 0.001); the minimum residual neuroretinal rim width showed a more pronounced difference (29.7 ± 8.2% vs 7.1 ± 3.9%, *P* < 0.001). The changes distributed predominantly towards the nasal direction in chON, contrasting the changes to the arcuate fibers in glaucoma. In conclusion, our results provide the first longitudinal evidence of true pathological cupping in chONcompared to photographically disease-free baseline. The marked difference in the residual minimum rim width reaffirms the importance of rim obliteration in the differential diagnosis between the two diseases.

## Introduction

Increased cupping of the optic disc is a characteristic sign of glaucoma but can also be observed in non-glaucomatous optic atrophies. Misdiagnosis, reported in 15 to 40% of cases^1–3^, may be critical due to the urgent need for surgical intervention in conditions such as compressive optic neuropathy (CON).

Previous literature has reported a large C/D ratio in CON^4–10^, and milder increase in cupping than in glaucoma^2^. Nevertheless, distinguishing between CON and glaucoma based on optic disc morphology still presents a significant challenge. It is likely because the precise patterns and extent of the cup changes in CON are unknown. Not much is known about progressive changes in CON relative to baseline healthy optic discs. Additionally, the broad normal range in the cup-to-disc (C/D) ratio complicates differential diagnosis further^11^.

In this study, we used a novel study design to investigate the absolute change from healthy to overt optic neuropathy. Fundus photographs preceding the onset of optic neuropathy were used for the purpose. We aimed to provide the first longitudinal evidence of pathologically increased cupping in chiasmal lesion optic neuropathy (chON) and a quantitative comparison of the neuroretinal rim (NRR) changes between chON and glaucoma.

## Results

Of 90 eyes of 56 patients with chON, we excluded 2 eyes of 2 patients with media opacity, 11 eyes of 6 patients with progressive myopic tilt during the follow-up period, 8 eyes of 4 patients diagnosed with glaucoma before the onset of chON, 2 eyes of 1 patient with a history of optic neuritis, and 36 eyes of 22 patients without final optic nerve damage. Of 38 eyes of 35 patients with glaucoma, we excluded 1 eye of 1 patient with disc contour changes due to the development of peripapillary staphyloma during the follow-up period, 1 eye of 1 patient with central retinal artery occlusion, and 3 eyes of 3 patients with media opacity.

Finally, 31 eyes of 21 patients with chON (chON group) and 33 eyes of 30 patients with glaucoma (glaucoma group) were included in the analysis. The chON group comprised 6 eyes of 3 patients with craniopharyngioma, 4 eyes of 3 patients with meningioma, 3 eyes of 2 patients with Rathke’s cleft cyst, and 18 eyes of 13 patients with pituitary adenoma.

### Clinical characteristics of patients

Table 1 summarizes the demographic and clinical characteristics of patients with chON and glaucoma. The visual field (VF) mean deviation was significantly worse in the chON group than in the glaucoma group. However, the PSD exhibited no significant difference, in line with the more focal VF damage observed in glaucoma than in chON. The interval between the baseline and final photographs were longer in glaucoma than in chON. Other variables were not different between the two groups.

**Table 1:** Clinical characteristics of patients with chON and glaucoma.

	chON	Glaucoma	P*
N (eyes)	31	33	
Age (years)	60.1 ± 14.0	64.9 ± 7.3	0.086
Refractive error (diopters)	− 1.03 ± 2.59	− 1.17 ± 1.92	0.846
Final VF mean deviation (dB)	− 11.81 ± 10.14	− 3.10 ± 3.47	< 0.001
Final VF pattern standard deviation (dB)	7.67 ± 5.77	5.79 ± 4.28	0.198
Final intraocular pressure (mmHg)	16.7 ± 2.7	15.8 ± 4.1	0.460
Fundus photograph interval (years)	8.1 ± 4.3	10.9 ± 2.7	0.010

*chON* chiasmal lesion optic neuropathy, *VF* visual field, *dB* decibel.

*Generalized estimation equation.

### Qualitative changes in cup, vascular configuration, and pallor in chON

The interobserver agreement for cup size change, vascular configuration change, and pallor were 0.871 (*P* < 0.001), 0.914 (*P* < 0.001), and 0.889 (*P* < 0.001), respectively.

In chON group, 23 eyes (74.2%) had changes in the contour of the cup in fundus photographs, 25 eyes (80.6%) had changes in vascular configuration, and 20 eyes (64.5%) had optic disc pallor developed at locations corresponding to RNFL damage. Twenty-six eyes (83.9%) had a significant change in either cup size or vascular configuration. Sixteen eyes (51.7%) had cup size change, vascular configuration change, and pallor. As expected, all eyes (100.0%) in the glaucoma group had changes in cup size and vascular configuration but no pallor.

When the vascular configuration change was separately analyzed in sectors of superior, inferior, nasal, and temporal, the distribution was different between the two groups. In the 25 chON eyes with vascular configuration change, 18 eyes had changes in the nasal sector, 3 eyes in the inferior sector, 1 eye in the temporal sector, and 1 eye in the nasal and inferior, nasal and superior, and nasal and temporal sectors, respectively. In glaucoma eyes, 12 eyes had changes in the inferior sector, 5 eyes in the superior sector, 14 eyes in the superior and inferior sectors, 1 eye in the inferior and nasal sectors, and 1 eye in the inferior and temporal sectors, respectively.

### Quantitative changes in optic disc morphology between chON and glaucoma

Table 2 shows the quantitative changes in cupping in the chON and glaucoma groups. The calculated disc size ratio was not different between the two groups (*P* = 0.890). The increase in cup size was significantly smaller in the chON group than in the glaucoma group, but the numerical difference was not dramatic (0.04 ± 0.04 vs. 0.10 ± 0.04, *P* < 0.001). In contrast, the minimum residual NRR width showed a marked difference between groups (29.7 ± 8.2% vs. 7.1 ± 3.9%, *P* < 0.001).

**Table 2:** Quantitative changes in optic disc morphology in patients with chON and glaucoma.

Quantitative parameters	chON	Glaucoma	P*
Disc size ratio^†^	0.36 ± 0.04	0.37 ± 0.04	0.890
Baseline C/D area ratio	0.30 ± 0.08	0.35 ± 0.07	0.012
Final C/D area ratio	0.34 ± 0.09	0.45 ± 0.07	< 0.001
Increase in C/D area ratio	0.04 ± 0.04	0.10 ± 0.04	< 0.001
Minimum residual NRR width (%)	29.7 ± 8.2	7.1 ± 3.9	< 0.001

*C/D* cup-to-disc, *NRR* neuroretinal rim, *chon* chiasmal lesion optic neuropathy.

*Generalized estimation equation.

^†^$$\mathrm{Disc\, size\, ratio}=\frac{\frac{\sqrt{(Disc\, area)}}{2\pi }}{(Disc\, to\, fovea\, distance)}.$$

### Topographic difference in optic disc morphology between chON and glaucoma

**Figure 1:**
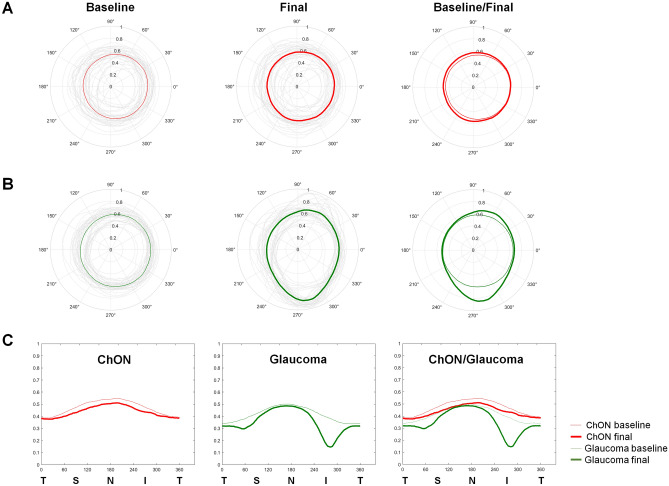
Polar plots of cup changes in chON and glaucoma. Polar plots of baseline and final percentage cup widths in the chON (**A**) and glaucoma (**B**) groups. The gray lines show individual values, and the red and green lines show the average values. (**C**) Predominantly nasal cupping in the chON group and superotemporal/inferotemporal cupping in the glaucoma group. All measurements were transformed to the left eye direction.

**Figure 2:**
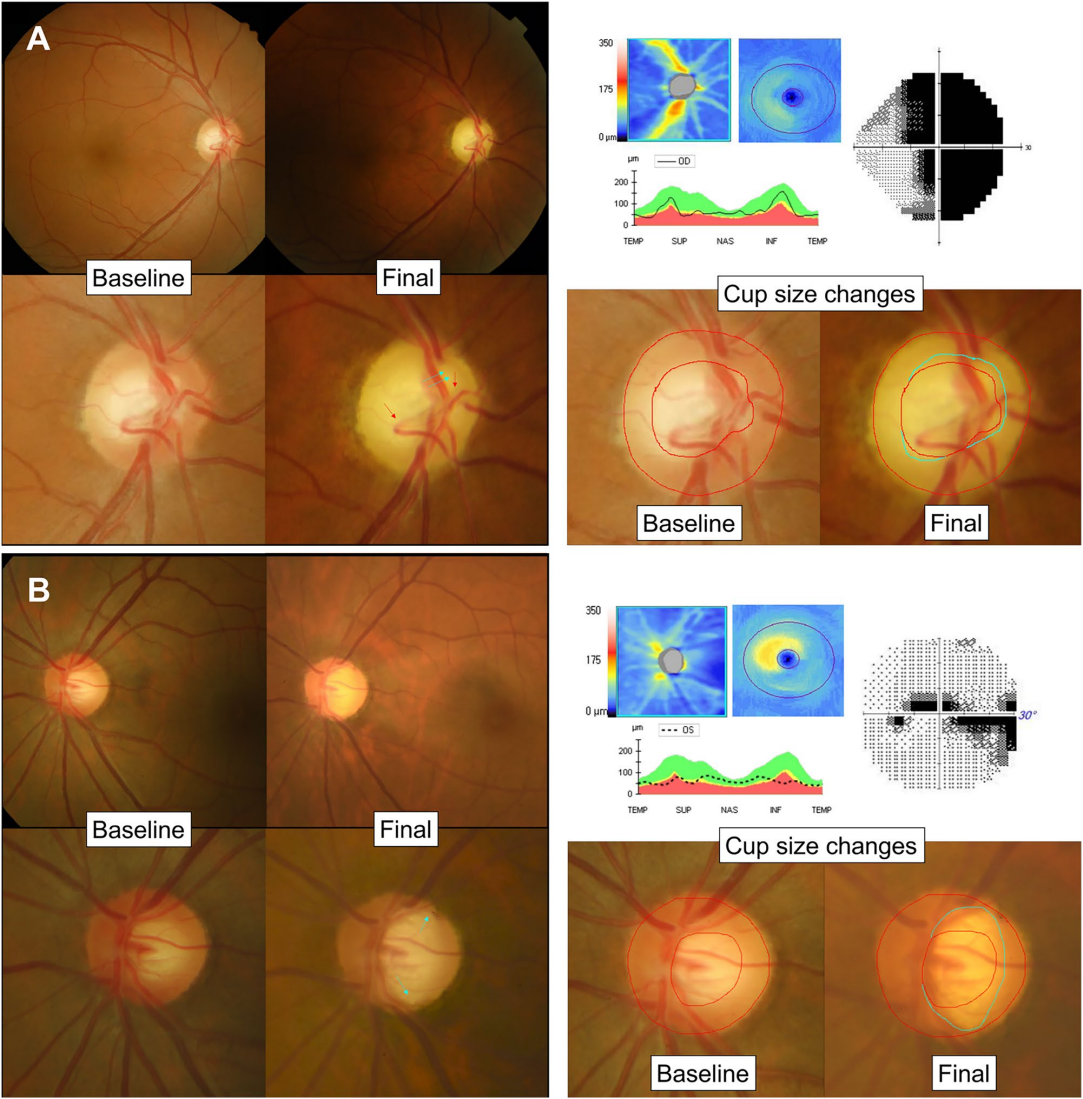
Representative cases of chON and glaucoma. (**A**) Case of chON. Compared to the baseline cup (red line), a diffuse subtle increase in cup size (blue line), accompanied by changes in cup contour (blue arrows) and vascular configuration (red arrows) were observed. (**B**) Case of glaucoma. Compared to the baseline cup (red line), a dramatic and focal increase in cup size (blue line) in the superotemporal and inferotemporal directions was observed. Note that the cup increase is so extensive that the cup margin touches the disc margin, and the residual width of NRR is therefore minimal. In both cases, the optic disc changes are in topographical correspondence with the loss of retinal ganglion cells, as shown in the OCT and VF test results.

The glaucoma group showed significant thinning of the NRR in the superotemporal and inferotemporal directions, whereas the chON group primarily exhibited NRR changes distributed towards the nasal side, as expected. Figure 1 shows the distributions of cup and NRR widths in polar graphs and temporal-superior-nasal-inferior-temporal (TSNIT) graphs, respectively. Figure 2 shows representative cases.

### Subgroup analysis between eyes with and without cup size change in chON

We observed no significant differences in the investigated parameters between the eyes with and without cup size change in chON (Table S1).

## Discussion

Among the numerous studies reporting the increased cupping in CON, most were conducted in cross-sectional designs^4–10^. Without the absolute change from the baseline, we may not be certain of true pathologic changes in cup.

Therefore, we attempted a novel study design to identify the changes of the cup compared to baseline photographically disease-free baseline optic discs. Our findings provide the first direct evidence of pathologically increased cupping in chON. We observed that over 70% of eyes with chON demonstrated a detectable increase in cup size. Our results confirm that the cross-sectionally demonstrated large cup in chON represents a true pathologic increased cupping associated with chON.

Our results also provide quantitative results. The increase in C/D area ratio was subtle (approximately 5% increase) in CON. This mild change explains previous study results comparing eyes with CON to normal controls^4,8,10,12^. For example, the mean C/D ratio in the CON and control groups was 0.29 ± 0.09 vs. 0.30 ± 0.12 using color photographs^12^, and 0.48 vs. 0.44 using Heidelberg retinal tomography^10^. These minimal differences may be potentially attributable to the subtlety of the changes. In addition, given our data, the large C/D ratios observed in CON in previous studies (e.g., 0.8–0.9)^4–7^ could have been partly due to pre-existing glaucoma^7,9^ or physiologic large cup; Radius and Maumenee reported cases with features of glaucomatous cupping where the fellow eyes manifested large physiological cups^12^.

Moreover, our results confirmed the more profound excavation in glaucoma compared to CON^2,10^. However, the difference in the C/D area ratio changes (0.04 vs 0.10) may not be large enough to reliably differentiate CON from glaucoma. In contrast, we observed a stark contrast in the minimum residual percentage NRR width (29.7% vs 7.1%) between CON and glaucoma, showing a sharper contrast between CON and glaucoma than the overall C/D area ratio or the change from baseline. This is in line with the report that focal or diffuse obliteration of the NRR was 87% specific for glaucoma, and the NRR pallor was 94% specific for non-glaucomatous optic atrophy, suggesting these two signs as the most useful in making the distinction^1^. A near-total loss of the NRR and the cup touching the disc margin is highly indicative of glaucoma. Paying attention to the characteristics of remaining NRR would be helpful in the differential diagnosis, particularly than examining the C/D ratio alone.

Our results also confirm the difference in topographic direction of cup change between glaucoma and chON. In our study, the cup changes were predominantly distributed in the nasal direction in the chON group compared to the inferotemporal and superotemporal changes in the glaucoma group (Fig. 2). Danesh-Meyer et al. also reported that the 3 o'clock temporal sector could help differentiate open-angle glaucoma from CON^10^.

Nevertheless, not all eyes exhibited detectable changes in cup size in chON in our study. We could not identify the relevant factors. The degree and topographical pattern of optic nerve damage, original configuration of the NRR, disc size, cup depth, study interval, and tumor characteristics are possible factors associated with the changes in cupping. Also for the eyes who did not exhibit significant vascular change, we consider the subtle damage and absence of the vessels exactly overlying the affected regional NRR area may have influenced on the results. Further studies are warranted on this issue.

Burgoyne discussed that non-glaucomatous optic neuropathies demonstrate predominantly prelaminar cupping^13^. We used fundus photographs, which cannot reveal information about the deep ONH characteristics such as changes in the LC. Only the superficially detectable changes could be analyzed, and thus, we considered the cupping in our study would largely represent the superficial components of the cupping and therefore, mostly prelaminar. The superficial flattening of the NRR have been previously mentioned in optic discs with pallor^14^. Further studies using OCT imaging in the deep ONH to evaluate the prelaminar and laminar components of cupping would be beneficial to better understand the different cup changes.

The different underlying mechanism of cupping between CON and glaucoma is unknown. Interestingly, the prelaminar glial cell reactions differ between CON and glaucoma. The glial cells are disorganized^15^ and ultimately disappear in the prelaminar area in glaucoma^16^. In contrast, the prelaminar glial cells in CON remains, maintaining their columnar configuration; they are compressed, resulting in decreased width of the NRR^17^. Further studies are warranted to identify the mechanism of different cupping between optic neuropathies.

This study had several limitations. First, the study was retrospective, the sample size was small, and we had no OCT results. However, data such as ours are rare, and a prospective design performing OCT in healthy status without need for disease evaluation is hardly possible. If possible, further studies using OCT would be helpful to understand the detailed prelaminar and laminar changes in a three-dimensional pattern, including the cup depth. Second, pallor was evaluated subjectively and analyzed with different instruments and pupil status between the baseline and the final examinations. During all examinations, meticulous attention was given to controlling light exposure, focus, and the framing of photographs to ensure consistent high-quality. Repeat examinations were conducted until satisfactory results are achieved. Moreover, the complete set of serial photographs was examined to account for variations due to inter-examination differences in photography. These efforts might have resulted in the near perfect interobserver agreement; however, it should be noted that the presence of pallor could also be affected by the methods in photography. Third, the method of cup margin delineation may be considered subjective and can be affected by different photograph conditions as well. We paid particular attention in obtaining an accurate estimate on the contour of the cup wall considering all possible clues provided in the photographs; the color, shades, connectivity to the adjacent structures, and vessels were comprehensively evaluated. Nevertheless, the delineation from fundus photographs may not indicate the true change in the 3D thickness distribution of the NRR, and the limit should be noted. However, at least, changes in the cup contours were the main focus of this study. We used simultaneous real-time flicker analysis, which provides a more sensitive detection of differences than conventional side-by-side comparisons and improves inter-grader agreement compared to other forms of optic disc assessment. Therefore, while the delineation may not be accurate in absolute measures, we consider that the result of difference from the baseline evaluated under consistent methods may provide useful insights, especially for results with marked difference as observed in our study. Fourth, the glaucoma group showed significantly milder damage than the chON group. The difference in observation period and VF loss should be considered in interpreting our results. It is even more important by the fact that no evidence other than photographs were available for the baseline. To compare the NRR changes under a given degree of optic nerve damage, it would be best to match the period and the degree of optic nerve damage occurred within it. However, in this study, we could not match them largely because glaucoma is a slowly progressive chronic disease and to obtain a case with baseline and advanced visual loss like those in severe chON, it would require decades of period. In contrast, chON may progress rapidly depending on the characteristics of the causative tumor. Nevertheless, we speculate that our results may still provide useful insights in clinical usage by expanding the results from regional to broader NRR areas. That is, the rim thinning in glaucoma is focal in the early stage but expands with disease progression. The observed difference in NRR thinning in this study, as shown by the minimum remaining NRR width, may be similarly maintained in eyes with further damage. Therefore, the observed regional difference in NRR may remain only with broader areas in optic discs between the two diseases.

In conclusion, we provide the first longitudinal evidence of pathologically increased cupping in chON from the photographically disease-free baseline, and quantitatively showed the significantly milder and more nasally distributed NRR thinning in chON compared to glaucoma. The marked difference in the residual minimum NRR width reaffirms the importance of rim obliteration in the differential diagnosis between the two diseases.

## Methods

This was a retrospective longitudinal study. The medical records of patients who visited the Neuro-ophthalmology and Glaucoma Department of the Samsung Medical Center (Seoul, South Korea) between April 2016 and March 2023 and underwent their earliest ophthalmologic examination during health screening at the Samsung Medical Center Health Promotion Center between January 2008 and October 2020 were reviewed. This study followed all guidelines for experimental investigation in human subjects, was approved by the Samsung Medical Center Institutional Review Board (#2023-04-076) and was conducted in accordance with the tenets of the Declaration of Helsinki. The Samsung Medical Center Institutional Review Board waived informed consent because of the absence of harm to subjects and the retrospective nature of the study design.

### Eligibility criteria

We included patients with CON and glaucoma who had undergone health screening examinations including fundus photographs. The fundus photographs had to be taken at least one year prior to the onset of optic neuropathy. The clinical diagnosis of chiasmal compression in patients with CON was based on preoperative VF defects and/or decreased visual acuity and magnetic resonance imaging (MRI) evidence of mass compression of the optic chiasm. All patients underwent transsphenoidal resection. Glaucoma was diagnosed based on signs of glaucomatous optic nerve damage (i.e., NRR notching or thinning or localized retinal nerve fiber layer [RNFL] defects) and a glaucomatous VF defect, defined as a defect conforming to one or more of the following criteria: (1) glaucoma hemifield test results outside normal limits, (2) at least three adjacent abnormal points with a *P* < 0.05 probability of being normal and at least one of these being abnormal with a *P* < 0.01 probability by pattern deviation, or (3) a pattern standard deviation (PSD) of *P* < 0.05, confirmed on two consecutive reliable tests.

We excluded patients with: (1) CON with pre-diagnosed glaucoma before the onset of CON; (2) progression of myopic optic disc changes during the follow-up period; (3) other concomitant ocular diseases (e.g., optic neuritis); and (4) poor-quality fundus photographs due to media opacity.

### Patient evaluation

All participants underwent visual acuity assessments, refraction tests, slit-lamp biomicroscopy, tonometry, dilated stereoscopic examinations of the optic disc, color fundus photography (TRC-50DX; Topcon Medical System, Inc., Oakland, New Jersey, USA), spectral-domain optical coherence tomography (OCT) of Spectralis OCT (Heidelberg Engineering, Heidelberg, Germany) or Cirrus HD-OCT (Carl Zeiss Meditec AG, Jena, Germany), and standard automated perimetry (HFA model 640; 30-2 or 24-2 Swedish interactive threshold algorithm; Carl Zeiss Meditec, Inc, Dublin, CA). A non-mydriatic fundus camera (Topcon TRC-NW; Topcon Corporation, Tokyo, Japan) was used for the health screening examination.

### Definition of the photographically disease-free baseline

We evaluated the baseline fundus photographs for any abnormalities in optic disc cupping, neuroretinal rim thinning, disc hemorrhage, or localized RNFL defect. The highly pigmented fundus in Asian eyes aided in discerning any subtle abnormalities in RNFL^18,19^. With the absence of any ocular pathology, we graded the fundus photograph normal and assumed it as presumably disease-free baseline. Moreover, the baseline photographs had to be documented at least one year before the referral or onset of subjective visual symptoms related to the optic neuropathy.

### Overlay image production for flicker analysis

**Figure 3:**
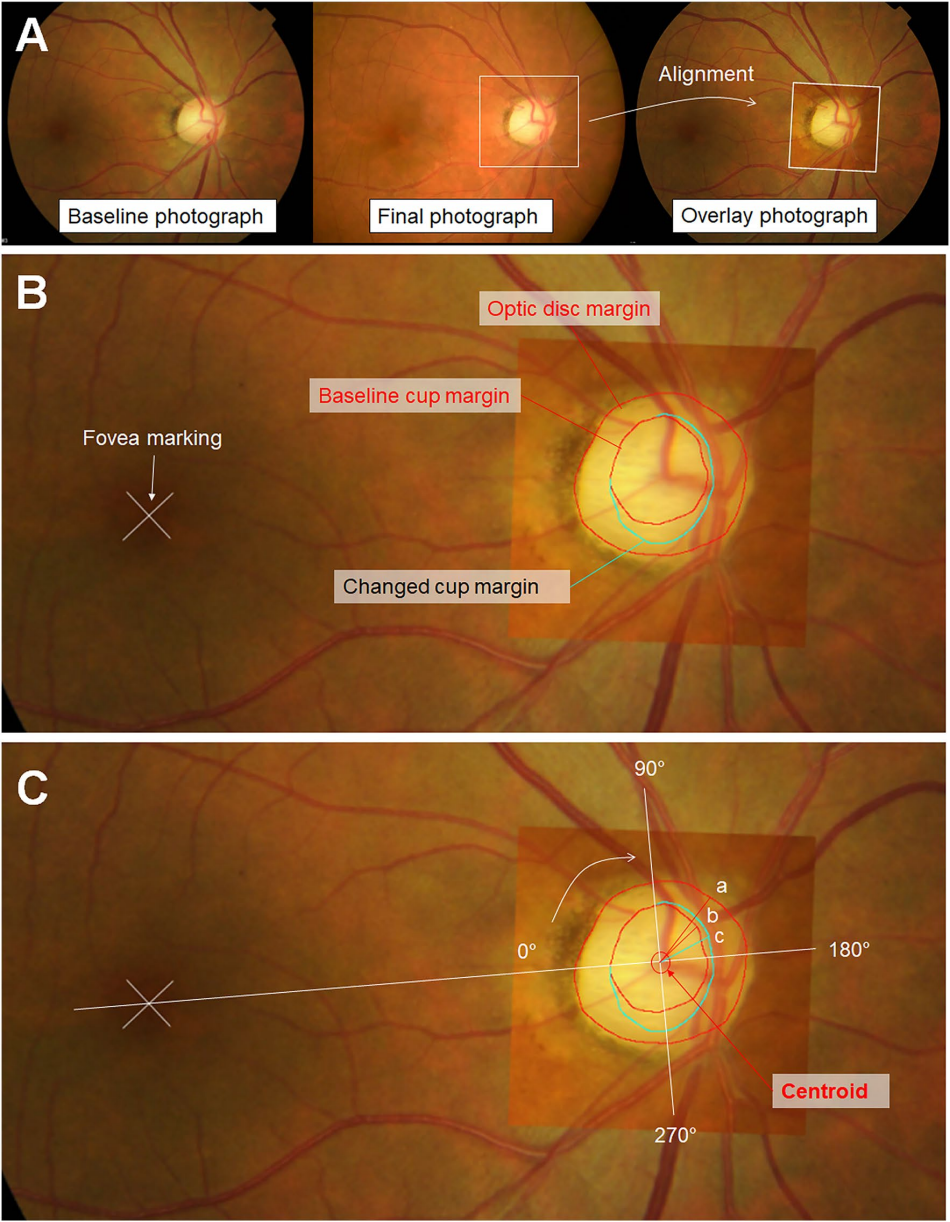
Evaluation of the optic disc and cup changes. (**A**) An overlay image for each eye by superimposing the cropped optic disc area of the fundus photograph taken at the final follow-up onto the baseline fundus photograph, aligned by the vascular landmarks. (**B**) Manual delineation of the cup and disc margins and location of the fovea. Flicker analysis was performed before the delineation of the changed cup margin in every measurement. (**C**) The radial widths of the disc margin (a), baseline cup (b), and final cup (c) from the automatically allocated centroid of the optic disc were collected radially by 1°. The direction of the fovea on the disc-fovea axis was designated as 0° and increased up to 360° in the temporal-superior-nasal-inferior-temporal (TSNIT) order.

We produced an overlay image for each eye by superimposing the cropped optic disc area of the fundus photograph taken at the final follow-up onto the baseline fundus photograph, aligned by the vascular landmarks (Fig. 3). We performed a real-time alternation flicker analysis of superimposed sequential images, known to provide more sensitive results than side-by-side comparisons for detecting changes in sequential images in terms of vessel position, color, and other cues for contour change^20,21^, using the layer function of Adobe Photoshop CC (Adobe Systems, Mountain View, CA, USA), to delineate the cup margin change in every measurement. We used the most recent photographs as the final photograph in both disease groups, assuming the maximum amount of optic nerve damage within the period. It was because the structural changes in optic disc and RNFL persists despite successful decompression surgery in eyes with chON, while a significant improvement may occur in the VF indices^22^. For glaucoma patients, since all eyes were monitored from a photographically normal baseline, the gradual progression of glaucoma led to optic disc cupping in the early to moderate stages for all eyes. Consequently, the most recent photograph was chosen to showcase the most pronounced and evident changes.

### Qualitative evaluation of the optic disc changes

We recorded the presence of the changes in cup size, vascular configuration, and optic disc pallor. We manually delineated the margin of the optic disc and cup, as well as the location of the fovea (Fig. 3). In assessing pallor, the overall brightness and contrast of retinal structures, including the optic disc, vasculature, and peripapillary atrophy area, were considered through a thorough review of all serial fundus photographs to address inter-examination variability.

The optic cup was determined based on its contour, with the outer margin taken to be the point where its wall met the plane of the disc surface^23^. The paths of vessels helped to define the contour of the NRR^23^. A significant vascular configuration change was defined as the difference in the course of the vessels overlying the NRR, which topographically corresponds to the RNFL loss. Specifically, we evaluated the displacement of vessels towards the outer part of the rim, aligning with regional thinning of the NRR and RNFL in the direction over which the vessel is positioned (e.g. a bayoneting sign).

Two independent readers blinded to the clinical information recorded the parameters, and a consensus was reached through discussion in discordant cases.

### Quantitative evaluation of the optic disc changes

In manual delineation of the contour of the cup (performed by E.J.L), we did not only depend on the color, such as redness of the NRR, but also on the overall shape of the NRR, the shades, and the relative connection and continuity to adjacent structures including vessels for the evaluation of contour. An imaginary wall of NRR was inferred from all the components including color, shades, as well as kinking of the overlying vessels as they climb on the cup wall and bend over the rim^24^. Other serial fundus photographs were also reviewed whenever necessary for accurate allocation of the edge of the NRR.

We developed a computer program utilizing commercial software (MATLAB ver. 7.3; The MathWorks, Inc., Natick, MA, USA). The program detected the manual delineation and automatically calculated the C/D area ratio. Radial widths of the disc and cup were computed from the automatically allocated centroids of the optic disc. The measurements were executed radially by 1°, designating the direction of the fovea on the disc-fovea axis as 0° and increasing up to 360° in the TSNIT order. The percentage cup width was calculated by dividing the cup width by the distance from the centroid to the disc margin in each measured direction. The percentage NRR width was calculated using the same method. In addition, we calculated the disc size ratio as follows to estimate the approximate size of the optic disc.

$$\mathrm{Disc\, size\, ratio}=\frac{\frac{\sqrt{(Disc\, area)}}{2\pi }}{(Disc\, to\, fovea\, distance)}$$

### Statistical analysis

Interobserver agreement for the presence of vascular configuration changes and optic disc pallor was assessed by Cohen’s kappa. Clinical characteristics were compared between the CON and glaucoma groups using a generalized estimation equation to consider the possible associations between both eyes of one patient. Statistical significance was set at *P* < 0.05. All statistical analyses were performed using SPSS version 23.0 (SPSS Inc., Chicago, IL, USA).

### Supplementary Information

Supplementary Table 1.

## Data Availability

The datasets used and/or analyzed during the current study available from the corresponding author on reasonable request.
