# Supplementary material for: Longitudinal changes in optic disc cupping from the baseline in chiasmal lesion optic neuropathy and glaucoma
Source: Sci Rep. 2024 Apr 17;14:8889. doi: 10.1038/s41598-024-59419-3 (PMC11024126; doi:10.1038/s41598-024-59419-3)
Supplement: Supplementary file 1 — Supplementary Table 1. [file 41598_2024_59419_MOESM1_ESM.docx]

**Supplementary Material**

**Table S1. Characteristics of eyes with and without cup size changes in chON**

|  | Without cup size change | With cup size change | *P*^*^ |
| --- | --- | --- | --- |
| N (eyes, %) | 8 | 23 | NA |
| Age at diagnosis (years) | 68.6 ± 8.3 | 57.1 ± 14.5 | 0.070 |
| Diagnosis (eyes) | Pituitary adenoma (5)  Craniopharyngioma (2)  Rathke’s cleft cyst (1) | Pituitary adenoma (13)  Craniopharyngioma (4)  Rathke’s cleft cyst (2)  Meningioma (4) | 0.638^†^ |
| Refractive error (D) | 0.03 ± 2.40 | -1.41 ± 2.60 | 0.220 |
| Final VF MD (dB) | -8.48 ± 8.19 | -12.97 ± 10.66 | 0.259 |
| Final VF PSD (dB) | 6.80 ± 5.45 | 7.98 ± 5.97 | 0.665 |
| Baseline C/D area ratio | 0.26 ± 0.11 | 0.31 ± 0.07 | 0.216 |
| Final C/D area ratio | 0.26 ± 0.11 | 0.36 ± 0.08 | 0.005 |
| C/D area ratio difference | 0.00 ± 0.00 | 0.06 ± 0.03 | NA |
| Disc size ratio | 0.37 ± 0.04 | 0.36 ± 0.04 | 0.506 |
| Fundus photograph interval (years) | 7.1 ± 4.9 | 8.5 ± 4.0 | 0.449 |

chON=chiasmal lesion optic neuropathy; D=diopters; VF=visual field; MD=mean deviation; PSD=pattern standard deviation; dB=decibel; C/D=cup-to-disc; NRR=neuroretinal rim; NA=not applicable.

^*^ Generalized estimation equation except for the diagnosis.

^†^ Pearson’s chi-square test.
